# Comparison of δ^13^C and δ^15^N of ecologically relevant amino acids among beluga whale tissues

**DOI:** 10.1038/s41598-024-59307-w

**Published:** 2024-05-15

**Authors:** Cory J. D. Matthews, Emma A. Elliott Smith, Steven H. Ferguson

**Affiliations:** 1 Fisheries and Oceans Canada, 501 University Crescent, Winnipeg, MB Canada; 2 National Museum of Natural History, Smithsonian Institution, Washington, DC USA

**Keywords:** Carbon, Compound-specific stable isotope analysis, *Delphinapterus leucas*, Essential, Glutamic acid, Nitrogen, Phenylalanine, Trophic, Biochemistry, Ecology

## Abstract

Ecological applications of compound-specific stable isotope analysis (CSIA) of amino acids (AAs) include 1) tracking carbon pathways in food webs using essential AA (AA_ESS_) δ^13^C values, and 2) estimating consumer trophic position (TP) by comparing relative differences of ‘trophic’ and ‘source’ AA δ^15^N values. Despite the significance of these applications, few studies have examined AA-specific SI patterns among tissues with different AA compositions and metabolism/turnover rates, which could cause differential drawdown of body AA pools and impart tissue-specific isotopic fractionation. To address this knowledge gap, especially in the absence of controlled diet studies examining this issue in captive marine mammals, we used a paired-sample design to compare δ^13^C and δ^15^N values of 11 AAs in commonly sampled tissues (skin, muscle, and dentine) from wild beluga whales (*Delphinapterus leucas*). δ^13^C of two AAs, glutamic acid/glutamine (Glx, a non-essential AA) and, notably, threonine (an essential AA), differed between skin and muscle. Furthermore, δ^15^N of three AAs (alanine, glycine, and proline) differed significantly among the three tissues, with glycine δ^15^N differences of approximately 10 ‰ among tissues supporting recent findings it is unsuitable as a source AA. Significant δ^15^N differences in AAs such as proline, a trophic AA used as an alternative to Glx in TP estimation, highlight tissue selection as a potential source of error in ecological applications of CSIA-AA. Amino acids that differed among tissues play key roles in metabolic pathways (e.g., ketogenic and gluconeogenic AAs), pointing to potential physiological applications of CSIA-AA in studies of free-ranging animals. These findings underscore the complexity of isotopic dynamics within tissues and emphasize the need for a nuanced approach when applying CSIA-AA in ecological research.

## Introduction

Consumer tissue stable isotope (SI) composition reflects that of their diet, with some offset due to isotopic fractionation during metabolism^1,2^. Consumer SI composition is therefore the result of cumulative alterations (i.e., during previous trophic steps) to baseline food web SI composition, which itself varies as a function of underlying biogeochemical processes (e.g., carbon and nitrogen sources, fixation pathways^3,4^). Ecological applications have made use of both trophic and baseline SI variation, inferring consumer trophic position primarily from ratios of naturally occurring stable nitrogen isotopes (δ^15^N), given diet-consumer isotopic fractionation typically exceeds baseline variation, and inferring habitat use (e.g., pelagic vs. benthic^5^) and largescale distribution from stable carbon isotope ratios (δ^13^C), given relatively small trophic ^13^C fractionation compared to baseline δ^13^C variation.

There are instances, however, in which baseline SI variation (both spatial and temporal) can confound ecological interpretations of consumer SI values. For example, bulk phytoplankton δ^13^C values at a single site can vary by > 10 ‰ over the course of a single year due to inorganic nutrient availability associated with upwelling, leading to overlapping δ^13^C values with benthic macroalgae and thus limiting the effectiveness of bulk δ^13^C analysis for determining carbon sources^6^. Similarly, isotopically distinct nitrogen sources and different primary producer communities impart ocean basin-scale δ^15^N variation that can range from 5–10 ‰^3^, exceeding typical trophic ^15^N enrichment of 2–5 ‰^7^. Bulk δ^15^N measurements alone cannot be used to partition trophic from baseline variation.

Amino acids (AAs) are a major conduit of carbon and nitrogen within organisms and food webs^8^, with each containing a carboxyl (−COOH) and an amino (−NH_2_) functional group. Carbon isotopes of AAs vary according to the biochemical groupings of essential and nonessential^8,9^. Because only primary producers, microbes, and fungi can synthesize essential AAs (AA_ESS_), animal consumers must acquire them directly via consumption. As a result, the carbon backbones of AA_ESS_ remain largely intact as they move from producers throughout food webs, resulting in minimal alteration to their δ^13^C values^8,10–12^, although gut microbes can alter values prior to consumer uptake^13^ and synthesize AA de novo for use by the host under protein limiting conditions^14^. Metabolic diversity among different primary producer taxa (e.g., macroalgae vs. terrestrial plants) imparts unique combinations of individual AA_ESS_ δ^13^C values that act as ‘fingerprints’ that can be detected at higher trophic levels^8,9,15^ and used to track consumer food sources and habitat use^16–19^.

Nitrogen isotope values (δ^15^N) vary bimodally between ‘source’ and ‘trophic’ AAs^20^, which show some overlap but are not completely aligned with essential and nonessential AAs. Amine bonds of source AAs are not broken during their dominant metabolic pathways, such that their δ^15^N values are largely conserved with each trophic step and therefore reflect baseline food web δ^15^N set by primary producers^21–23^. Trophic AAs, in contrast, enter metabolic pathways involving deamination reactions with associated kinetic isotopic fractionation that leads to collective ^15^N enrichment (increasing δ^15^N) with each trophic step^24^. Referencing trophic AA δ^15^N against the consumer’s own source AA δ^15^N values can therefore serve as an internal gauge of trophic position, while source AA δ^15^N alone serves as a baseline proxy for assessing animal movements and distributions (e.g., ^25^). However, considerable deviations of trophic and source AA δ^15^N patterns in marine mammals^26–28^ from conventional patterns established by earlier CSIA-AA studies (21,22,23) can result in inaccurate trophic position estimates for these taxa^27^, and highlight the need for a more refined understanding of amino-acid metabolism and isotopic alteration.

Mechanisms causing unexpected variation in consumer AA-specific δ^15^N and δ^13^C are, in many cases, not well understood^17,26,29–31^. An understudied but potential source of variation is tissue-specific AA-specific isotopic fractionation. Direct assimilation of some AAs from diet to tissue has little to no impact on their SI values (e.g., δ^13^C of AA_ESS_ or δ^15^N of source AAs^32^). However, reactions involving the breaking of C or N bonds, such as biosynthesis of non-essential AAs (AA_NESS_) using C from other AAs (as well as carbohydrates and lipids; see ^33,34^), or biosynthesis of trophic AAs via transamination and deamination of other AAs^32^, can impact the rates at which body AA pools are consumed and fractionated isotopically. Moreover, all AAs are subject to a suite of catabolic reactions when they exceed amounts required for protein synthesis, which can lead to isotopic enrichment of remaining AA pools (e.g.,^35^). Tissue-specific differences in AA composition, growth, and metabolic rate can therefore lead to differential rates of drawdown from body AA pools, particularly because AAs are often physically and metabolically compartmentalized in closed pools within and between tissues^32,36,37^. Schmidt et al.^38^, in one of the few studies to address this topic, found variations in AA δ^15^N between euphausiid (*Euphausia superba*) tissues predominantly reflected different rates of protein synthesis vs. degradation and size of AA pools.

Questions regarding consumer SI dynamics are best resolved using controlled diet studies (e.g.,^13^). However, studies involving captive marine mammals are restricted to relatively non-invasive sampling of tissues such as blood during routine examinations. The range of tissues typically examined in SI studies of wild animals is constrained to opportunistic sampling of dead animals in captivity, which often results from illness and thereby casts doubt on the generalization of SI results. Therefore, to evaluate how tissue-specific factors (e.g., protein composition, growth/turnover rate) might impact AA-specific SI values in marine mammals, we opted for an alternative approach. Instead of relying on captive subjects, we conducted a comparative analysis of δ^13^C and δ^15^N values for 11 AAs in skin, muscle, and dentine sampled from wild beluga whales (*Delphinapterus leucas*) hunted by Inuit. Skin, muscle, and teeth are commonly used in isotopic studies of marine mammals, primarily due to their routine accessibility during necropsies of stranded animals. Additionally, skin is easily biopsied from free-ranging animals. These tissues differ in their major protein composition and isotopic turnover rates. Muscle is composed primarily of the myofibrillar proteins actin and myosin^34^, while skin and the organic component of dentine in teeth consist primarily of collagen^40,41^. Skin and muscle represent large body protein reservoirs with turnover rates that reflect the balance between the rates of protein synthesis and breakdown. Dentine by comparison represents a much smaller overall protein pool with relatively slow, continuous deposition with no significant remodelling occurring afterwards^42^.

We hypothesized essential and source AAs would have the same δ^13^C and δ^15^N values, respectively, across tissues because neither carbon bonds of AA_ESS_ nor amine bonds of source AAs are altered during typical metabolism (but see^28,35^ regarding phenylalanine δ^15^N). In contrast, we hypothesized that δ^13^C and δ^15^N values of non-essential and trophic AAs may differ among tissues, potentially reflecting differential rates of de novo synthesis and drawdown of AA pools. Belugas represent an interesting study species in this respect since rapid skin proliferation during their annual molt^43^ could place relatively large demands on body AA pools over a brief, but intense, period of summer growth, in comparison to the much smaller amounts of collagen deposited continually in tooth dentine. Our primary objective was to determine whether tissue selection could contribute to error in ecological applications of compound specific stable isotope analysis of amino acids (CSIA-AA), particularly with respect to deviations of AA-specific SI patterns from assumptions caused by unforeseen physiological factors.

## Methods

### Sample collection and treatment

Skin, skeletal muscle, and mandibles were collected by Inuit hunters from each of seven beluga whales from Cumberland Sound, Baffin Island, Canada from June–August 1986–2008 as part of a long-running community-based sampling program. Cumberland Sound belugas were selected for this study because their restricted range^44^ should minimize the impact of spatial SI variation on SI composition of tissues with different turnover rates (see Discussion). Six of the seven whales were assumed to be sexually mature (> 12 yr. old^45,46^ and close to or at asymptotic adult size^47^;Table 1), which should also minimize the impact of variable growth rates on tissue SI incorporation (see^48^). All tissues were stored at –20°C immediately after collection.

**Table 1:** Sex, age (estimated from annual growth layer group counts), and body length (rostrum to tail notch) of the seven Cumberland Sound belugas (*Delphinapterus leucas*) for which compound specific stable isotope analysis of amino acids (CSIA-AA) was conducted on skin, muscle, and dentine.

DFO ID	Sex	Age	Length
ARPG86-02	M	12	359
ARPG-xx-1220	M	24	328
ARPG-xx-1238	F	39	427
ARPG-xx-1241	M	6	272
ARPG-xx-1281	M	14	366
ARPG-xx-1378	F	13	Not available
ARPG-xx-1382	M	38	Not available

Approximately 1 g portions of skin and muscle were excised from larger frozen samples and finely diced, freeze-dried, and lipid-extracted using a 2:1 chloroform:methanol mixture placed in a 30 °C water bath for 24 h. Samples were decanted and the process was repeated to ensure complete lipid removal, followed by drying under a fumehood for 24 h. One mandibular tooth from each whale, usually from position 2 or 5 on the left side, was sectioned longitudinally using a water-cooled diamond-edged saw blade. The entire core of exposed dentine was sampled using a micromill fitted with a 1-mm diameter carbide drill bit at a depth of 500 μm. Dentine was decalcified to isolate collagen for CSIA-AA (see^49^) using repeated 12 h washes in 12 M hydrochloric acid (HCl) at 4°C, followed by repeated rinses with distilled water. Collagen samples were then freeze dried without further treatment.

### CSIA-AA

Approximately 3 mg of each tissue sample was acid hydrolysed in 6M HCl at 150 °C for 70 min under a N_2_ headspace and derivatized using methyl chloroformate^50,51^. The carbon and nitrogen isotopic compositions of derivatized AAs were measured by gas chromatography-combustion isotope ratio mass spectrometry (GC-IRMS) using a Trace Ultra GC gas chromatograph coupled to Thermo Delta V Plus through a GC IsoLink (δ^13^C: column: DB-23 [Agilent Technologies], 30 m, 0.25 mm O.D., 0.25 mm film; δ^15^N: column: DB-1301 [Agilent Technologies], 60 m, 0.25 mm O.D., 1 μm film). All samples were analysed in duplicate for both δ^13^C and δ^15^N, and two in-house pure AA mixtures previously calibrated to the international reference scales for δ^13^C (Vienna Pee Dee Belemnite [VPDB] carbonate) and δ^15^N (atmospheric N_2_ [Air]) were used in calibration and scale normalization procedures. A third AA mixture served as the primary quality control reference, and two well-characterized natural materials, baleen and fish muscle, served as secondary quality control references^51^. To account for exogenous carbon added to AAs during derivatization and kinetic isotope effects, correction factors were calculated for each methoxycarbonyl (MOC) AA ester^52^ using δ^13^C values of underivatized and derivatized reference AAs following protocols detailed in Walsh et al.^50^. Analyses were conducted at pH <  <  < 1 to ensure production of a single derivative (pyroglutamic acid^53^) that retains the δ^15^N of the original underivatized Glu^50,51,54^.

Carbon isotopes of the following 11 AAs were measured in muscle and skin: glycine (Gly), alanine (Ala), aspartic acid/asparagine (Asx), glutamic acid/glutamine (Glx), proline (Pro), threonine (Thr), isoleucine (Ile), valine (Val), phenylalanine (Phe), leucine (Leu), and methionine (Met). Glx and Asx refer to the AA mixtures produced during acid hydrolysis, when Glutamine (Gln) and asparagine (Asn) are converted to their respective acids, glutamic acid (Glu) and aspartic acid (Asp). Nitrogen isotopes of the same AAs were measured in all three tissues, except for Thr (not measured) and lysine (Lys) (measured). Mean standard deviations of repeated δ^13^C measures (n = 60) of reference compounds ranged from 0.38 to 1.71 ‰ (mean = 0.94 ‰); mean standard deviations of duplicate sample measures (n = 14) ranged from 0 to 1.63 ‰ (mean = 0.29 ‰). The same values for δ^15^N were 0.38 to 1.79 ‰ (mean = 0.76 ‰; n = 67) and 0.01 to 1.47 ‰ (mean = 0.40 ‰; n = 21), respectively.

### Data analysis

Anabolic and catabolic biochemical pathways link multiple AAs through interchangeable intermediates and end products, for both C^55^ and N^32^. We therefore performed a multivariate repeated measures analysis (i.e., repeated measures MANOVA), which takes potential correlations among multiple dependent variables into account. Amino acid δ^13^C and δ^15^N values were treated as multivariate dependent variables, with ‘tissue’ as the within-subject factor measured for each of the seven belugas (subjects). δ^13^C and δ^15^N values were modelled separately, as biochemical pathways involving breaking and formation of C and N bonds are largely independent^32,55^. p-values for the Modified ANOVA-Type Statistic (MATS) were based on parametric bootstrap resampling, and when the global null hypothesis of the multivariate analysis was rejected at alpha 0.05, we conducted univariate post-hoc comparisons of each AA δ^13^C or δ^15^N value by tissue type, with parametric bootstrap resampling and Bonferroni adjustment for multiple testing. All analyses were conducted in R^56^ using the MANOVA.RM package^57^.

### Ethics approval

Ethics approval was not required for this study as all samples were collected from legally hunted animals with the approval of the Pangnirtung Hunters and Trappers Organization.

## Results

### Carbon isotope ratios (δ^13^C) of muscle and skin AAs

**Figure 1:**
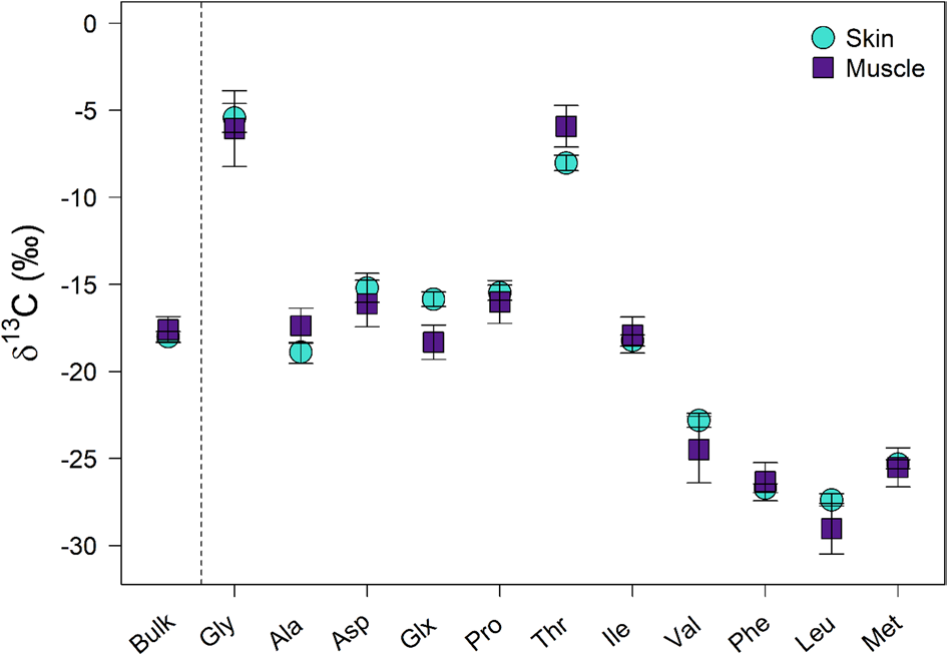
Mean δ^13^C values with standard deviation bars of 11 amino acids in beluga (*Delphinapterus leucas*) skin (turquoise circles) and muscle (purple squares). Mean bulk tissue values (lipid-extracted) are shown to the left of the dashed vertical line for reference.

Amino acid δ^13^C values differed between muscle and skin (Modified ANOVA-Type Statistic = 86.9, parametric bootstrap resampling p-value < 0.005; Fig. 1, Table 2). Univariate post-hoc tests did not detect significant differences in δ^13^C for nine of the 11 AAs (Ala, Asx, Gly, Ile, Leu, Met, Phe, Pro, and Val; Bonferroni-adjusted p = 0.07 to 1.00) between muscle and skin. δ^13^C of the non-essential AA Glx differed between muscle (− 18.3 ± 1.0 ‰) and skin (− 15.9 ± 0.4 ‰) (Bonferroni-adjusted p-value = 0.011; Fig. 1, Table 2). Threonine was the only essential AA for which δ^13^C differed significantly between muscle (− 5.9 ± 1.2 ‰) and skin (− 8.0 ± 0.4 ‰), with a Bonferroni-adjusted p-value of 0.033 (Fig. 1, Table 2).

**Table 2:**
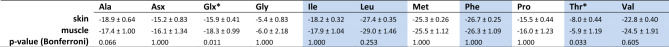
Mean (± SD) bulk tissue and amino acid specific δ^13^C values measured in skin and muscle of seven beluga whales (*Delphinapterus leucas*).

Results of univariate post-hoc tests are shown, with Bonferroni-adjusted p-values. Amino acids with significant δ^13^C differences (Bonferroni-adjusted p-value < 0.05) between skin and muscle are noted by *. Essential amino acids are indicated by shading.

### Nitrogen isotope ratios (δ^15^N) of muscle, skin, and dentine collagen AAs

**Figure 2:**
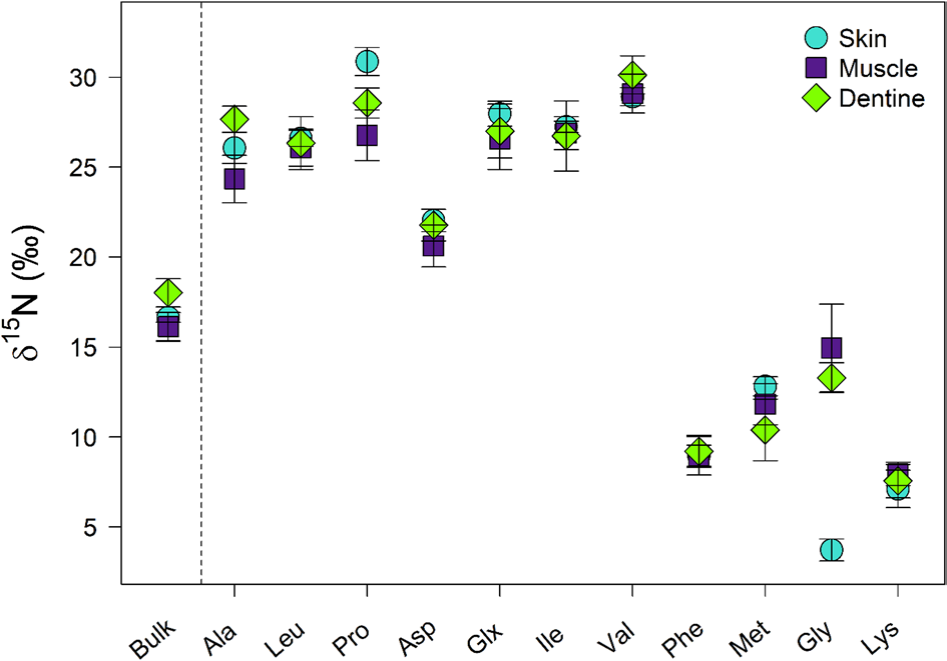
Mean δ^15^N values with standard deviation bars of 11 amino acids in beluga (*Delphinapterus leucas*) skin (turquoise circles), muscle (purple squares), and dentine (green diamonds). Mean bulk tissue values (lipid-extracted for both skin and muscle) are shown to the left of the dashed vertical line for reference.

Amino acid δ^15^N values differed among muscle, skin, and dentine collagen (Modified ANOVA-Type Statistic = 809.6, parametric bootstrap resampling p-value < 0.001; Fig. 2, Table 3). Univariate post-hoc tests did not detect significant differences in δ^15^N of the most ecologically relevant source (Phe) and trophic (Glx) AA pair (Bonferroni-adjusted p = 1.00 for both; Table 3). We also failed to detect significant differences in δ^15^N of Asx, Ile, Leu, Lys, Met, or Val among the three tissues (Bonferroni-adjusted p = 0.132 to 1.00; Table 3). δ^15^N values of the three remaining AAs, Ala, Gly, and Pro, differed among tissues (Bonferroni-adjusted p < 0.001; Table 3). Of note was the approximately 10 ‰ lower δ^15^N value of Gly in skin compared to muscle and dentine collagen, the largest range in SI values across tissues of any AA measured in our study (Fig. 2, Table 3).

**Table 3:**
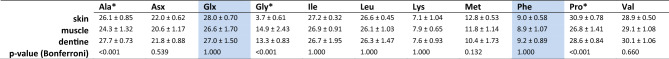
Mean (± SD) bulk tissue and amino acid specific δ^15^N values measured in skin, muscle, and dentine collagen of seven beluga whales (*Delphinapterus leucas*).

Results of univariate post-hoc tests are shown, with Bonferroni-adjusted p-values. Amino acids with significant δ^15^N differences (Bonferroni-adjusted p-value < 0.05) among skin, muscle, and dentine are noted by *. The most popular trophic-source AA pairing used in trophic position estimates (Glx and Phe, respectively) is indicated by shading.

## Discussion

Our prediction that δ^13^C of essential AAs and δ^15^N of source AAs would be similar across tissues was more or less borne out, supporting our hypothesis of direct routing of those AAs to tissues with no or minimal isotopic alteration. Moreover, the largely similar δ^13^C and δ^15^N of most non-essential AAs and trophic AAs among tissues points to possible direct routing of those dietary AAs into tissues, which would be more energetically efficient than de novo AA synthesis given the protein-rich diets of beluga whales (see^58^). Alternatively, similar δ^13^C of non-essential AAs and δ^15^N of trophic AAs among tissues could reflect largely similar biosynthetic pathways drawing C and N from shared AA pools^10,32^. Amino acids that differed isotopically among tissues, however, included both essential (Thr) and trophic (Pro, Ala) AAs, potentially making tissue selection relevant when interpreting CSIA-AA with ecological contexts.

Minimal offsets in isotopic compositions of AAs among tissues have been reported in other taxa, for example between domestic pig (*Sus domesticus*) muscle and bone collagen δ^13^C and δ^15^N^10^, southern sea otter (*Enhydra lutris nereis*) muscle, liver, and bone collagen δ^13^C^59^, coral polyp and skeletal protein δ^13^C and δ^15^N^60^, fish (*Lutjanus ehrenbergii*) muscle and otolith protein δ^13^C^16^, and fish (*Apogon semilineatus*) muscle and scale δ^15^N^61^. On the other hand, McMahon et al.^60^ attributed higher δ^15^N of trophic AAs in coral polyp tissue relative to skeletal proteins to higher protein turnover rates in the metabolically active polyp tissue. Interestingly, beluga skin generally had similar AA-specific SI values as muscle and dentine collagen, despite its rapid proliferation during the annual molt^43^. Isotopic fractionation is expected to be highest in metabolically active tissues, such as glandular tissue^38^ or liver^35,59,62^. These and other tissues and organs are sometimes collected from marine mammals during necropsy of recently hunted or stranded animals, and therefore comparison of additional tissues is warranted (see^59^).

Differences in carbon isotope composition detected for Glx (a non-essential AA) and Thr (an essential AA) among tissues are largely consistent with their synthesis or catabolism, associated with their specific roles in key metabolic processes. Differences in Glx δ^13^C between muscle and skin could potentially reflect incorporation of lipid-derived C via ketogenic pathways (synthesis from intermediaries of the tricarboxylic acid cycle). Newsome et al.^63^ found δ^13^C values of glutamate increased significantly in rodent (*Mus musculus*) muscle with increasing dietary lipid content, which reflected incorporation of lipid-derived carbon during protein synthesis. The lower δ^13^C values of Glx in beluga muscle relative to skin would be consistent with the incorporation of isotopically lighter lipid-derived C (and although not significant, the lower δ^13^C values of Asx, another ketogenic AA, in muscle are also consistent with this). Belugas consume high proportions of dietary lipids, and de novo AA synthesis from lipid-derived C may be energetically favorable, or even required given the high proportions of both AAs in muscle^63^. Belugas also exhibit seasonality in their diet^64,65^, and so it is also possible that muscle integrates periods of low food intake or fasting during which C inputs from catabolized blubber could contribute to protein synthesis. However, direct observations, stomach contents, and body condition indicate beluga feeding activity peaks during winter^64,65^, so the lower δ^13^C of muscle relative to skin would not be expected (as skin proliferates during the summer when feeding activity is thought to be reduced and would therefore also presumably incorporate lipid-derived C).

Threonine δ^13^C differences between muscle and skin were not anticipated because hr is an essential AA. Threonine catabolism in mammals proceeds primarily via two pathways initiated by either threonine dehydratase, which yields ammonia and α-ketobutyrate and ultimately propionyl-CoA (both glucogenic intermediates of the tricarboxylic acid cycle), or threonine dehydrogenase, which produces an unstable intermediate (2-amino-3-ketobutyrate) that can go on to produce glucose or additional enzymatic reaction to produce acetyl-CoA and glycine^66,67^. For a marine mammal with a high protein and high lipid diet, carbohydrates can be a limiting resource, and gluconeogenesis is likely necessary to meet glucose requirements (see also Discussion re: alanine below). Thr catabolism could thus lead to ^13^C enrichment of remaining Thr pools used for protein synthesis, particularly as muscle is more likely to integrate a long-term signal of Thr catabolism outside of the summer months, when increased foraging may shunt more Thr to gluconeogenesis than protein synthesis (see^68^).

From an ecological standpoint, δ^13^C variation in this and other studies (e.g.,^59,69^) suggest AA_ESS_ fingerprinting studies should use Thr with caution. We also note that the measurement error of CSIA-AA is relatively large compared to bulk SIA, for both C and N, and enhanced precision may reveal statistical differences. Moreover, while our restricted sample size is not uncommon in controlled diet studies with similar objectives to ours (e.g.,^13,59^), a larger sample size leading to improved statistical power may also reveal differences. Measured differences in Val and Leu δ^13^C of 1.7 and 1.6 ‰ between skin and muscle, while not statistically significant, could nevertheless be relevant within the context of underlying spatiotemporal variation. It therefore becomes crucial to assess these differences in relation to the magnitude of baseline δ^13^C variation to assess ecological significance. Consequently, Ile and Phe emerge as the only two AA_ESS_ with unequivocally similar δ^13^C among tissues in this study.

Ecological applications of AA-specific δ^15^N focus largely on trophic position estimation, and the impact of δ^15^N variation among tissues can be illustrated using the standard trophic position estimating equation^23^:

$$TP = \frac{{\delta^{15} N_{Tr} - \delta^{15} N_{Src} - \beta_{Tr - Src} }}{{TDF_{Tr - Src} }} + 1,$$

where δ^15^N_Tr_ and δ^15^N_Src_ are the paired consumer trophic and source AA δ^15^N values, typically Glx and Phe, respectively. β_Tr-Src_ is the initial difference in δ^15^N between the trophic-source AA pair in primary producers, and TDF_Tr-Src_ is the difference in fractionation of the trophic (Δ^15^N_Tr_) and source (Δ^15^N_Src_) AA with each trophic step. TDF_Glx-Phe_ has not been determined empirically for cetaceans; however, values of 4.3 ± 1.2 ‰ and 3.5 ± 0.4 ‰ have been determined in controlled diet studies of harbour seals (*Phoca vitulina*)^26^ and gentoo penguins (*Pygoscelis papua*^12^), respectively. The 1.4 ‰ range we find here in δ^15^N_Glx_ across tissues, although not statistically significant, results in a δ^15^N_Glx_–δ^15^N_Phe_ range of 17.6 ‰ (muscle) to 19.0 ‰ (skin), assuming δ^15^N_Phe_ is constant among tissues at ~ 9.0 ‰ (Table 3). That range of δ^15^N_Glx_–δ^15^N_Phe_ results in TP estimates of 3.3 (muscle) to 3.5 (skin) using the seal-derived TDF_Glx-Phe_, and 3.9 (muscle) to 4.3 (skin) using the penguin-derived TDF_Glx-Phe_ (see Matthews et al.^27^ for full equations). While the accuracy of CSIA-AA-derived TP estimates relative to beluga stomach contents has been discussed previously^27^, we find that additional error introduced by tissue selection is negligible. The differences of 0.2 and 0.4 between the estimates based on different tissues are within the range of propagated errors around CSIA-AA-derived TP estimates (0.3 to 0.6^27^). Similar δ^15^N values of Lys, a source AA that has been used as an alternative to Phe in trophic position calculations, in each of the measured tissues would also produce similar estimates independent of tissue choice.

Tissue-specific δ^15^N differences of other AAs, however, could impact trophic interpretations when using other trophic-source AA pairings, or averages across multiple AAs (e.g.,^30^). Proline, for example, has been proposed as an alternative to Glx in trophic position calculations^70^. Significant δ^15^N_Pro_ differences of up to 4 ‰ among tissues are comparable to TDF_Pro-Phe_ values in marine carnivores^71^, and thus would be influential in TP calculations based on that trophic-source AA pairing. Proline is one of the most abundant AAs in collagen, which in turn is the most abundant protein in the body^72^. Germain et al.^26^ postulated that variable trophic ^15^N enrichment of Pro in harbour seals *Phoca vitulina* had to do with its central role in the formation of collagen. By extension, differential rates of Pro synthesis could lead to the tissue differences we observed, as the two collagen-rich tissues in our study, dentine and skin, had higher δ^15^N values than muscle. Moreover, δ^15^N_Pro_ was highest in skin, which could reflect additional demands on Pro pools during a period of relatively high proliferation during the seasonal molt.

Lower δ^15^N values of alanine, also considered a trophic AA, in muscle relative to skin and dentine may have to do with its role in gluconeogenesis. Gluconeogenesis allows animals on protein-rich, low-carbohydrate diets (or alternatively, fasting) to maintain necessary glucose levels through conversion of AAs to pyruvate or tricarboxylic acid cycle intermediates for energy production^73,74^. In the glucose-alanine cycle, or Cahill cycle, N from catabolised AAs in muscle is transaminated to pyruvate to form alanine, which is then transported from the muscle to the liver and converted to glucose^75^. Isotopic fractionation would favor transamination of ^14^N, producing Ala that is depleted in ^15^N relative to catabolized AAs (see ^76^). Given that muscle cells are the primary site of AA degradation and Ala formation in the glucose-alanine cycle, ^15^N-depleted Ala would be expected in muscle relative to other tissues, and is consistent with predictions that gluconeogenesis would contribute to the overall metabolism of belugas that feed primarily on fish.

Glycine δ^15^N values exhibited the largest range of any AA across tissues in our study, exceeding 10‰. Notably, δ^15^N of Gly in skin was not just lower than Gly in muscle and dentine, but was also considerably lower than the δ^15^N of *all* other measured AAs in skin (Fig. 2, Table 3). Pathways of endogenous synthesis of glycine in rats and pigs includes transamination of the amine group from glutamate, as well as via the threonine dehydrogenase pathway^32,67,77^. Production of ^15^N-depleted glycine would be expected from both pathways, as isotopic fractionation during transamination of glutamate would favor ^14^N, and the amine group of threonine, which, unlike other AAs, exhibits serial ^15^N depletion with trophic level^78^, is retained during conversion to Gly. Conversion of glutamate/serine and threonine combined, however, accounts for only about 12% of endogenously synthesized Gly in pigs^67,68^. This is also inconsistent with observed Thr δ^13^C values, which would be expected to be higher in skin during periods of high Thr catabolism, and not muscle as observed (see above Discussion re: threonine catabolism). Meeting presumably high requirements of Gly, the most abundant AA in collagen^72^, during the seasonal skin molt could therefore involve any of the large proportion of substrates for endogenous Gly synthesis that remained unidentified and possibly account for the considerably lower δ^15^N values observed in skin. Gly had initially been designated as a source AA, but meta-analyses of Gly δ^15^N across a range of taxa (e.g.,^17,71^) showed the AA to be too variable to be used reliably as such, a conclusion further supported by our results showing an equally high, if not greater, degree of variation among tissues within a single species.

While our study of wild belugas provided access to tissues not typically sampled from captive marine mammals, we acknowledge confounding factors, notably uncontrolled variables such as seasonal diet shifts between isotopically distinct prey and movements among isotopically distinct habitats. These factors could lead to isotopic variation among consumer tissues as a function of growth/turnover rate, rather than factors hypothesized in this study. For CSIA-AA studies, this is complicated by the considerable variation in the isotopic equilibration rates of individual AAs, which can vary by over an order of magnitude^13,79^. Diet shifts and/or baseline SI variation could therefore lead to isotopic mismatches among tissues due simply to differential incorporation rates of individual AAs. While belugas migrate seasonally and are known to have seasonal variation in diet^64,65^, the restricted geographic range of this beluga population within Cumberland Sound^44^ should minimize impacts of these factors. The consistency of δ^15^N of most AAs across tissues with a widely varying turnover or deposition rates in fact suggests seasonal movements and/or variation in diet had negligible impacts on our results, since baseline SI shifts would similarly affect all AAs, and trophic shifts would affect most.

Considering the essential and source AAs for which we detected significant SI differences among tissues, ecological interpretations based on AA-specific δ^13^C or δ^15^N could be subject to potential biases. Potential error introduced by tissue selection, however, depends on the magnitude of the observed differences relative to baseline variation for δ^13^C, or to the magnitude of trophic discrimination for δ^15^N, which is variable in higher marine consumers^27,71^ and, for some AAs at least, appears to be tissue-specific. While a comprehensive assessment of AA-specific SI patterns from a physiological or biochemical point of view is beyond the scope of this study, it is noteworthy that the observed differences among tissues are largely consistent with catabolic or biosynthetic pathways associated with key physiological processes, thus highlighting potential novel applications of CSIA-AA in studies of animal physiology and biochemistry. Despite current uncertainties in absolute trophic position estimates using AA-specific δ^15^N [e.g., 27], these applications could involve assessing the relative importance of gluconeogenesis and ketogenesis in the nutrition of free-ranging animals – a task that is otherwise challenging due to the limitations of conventional methods. Insights thus gained from CSIA-AA could shed light on animal physiology and biochemistry in natural, ecological contexts.

## Data Availability

The datasets analysed for this study are available from the corresponding author upon request.
